# The correlation between epicardial fat thickness and longitudinal left atrial reservoir strain in patients with type 2 diabetes mellitus and controls

**DOI:** 10.1186/s13089-023-00338-1

**Published:** 2023-09-12

**Authors:** Maryam Nabati, Farideh Moradgholi, Mahmood Moosazadeh, Homa Parsaee

**Affiliations:** 1 Professor of Cardiology, Fellowship of Echocardiography, Department of Cardiology, Faculty of Medicine, Cardiovascular Research Center, Fatemeh Zahra Teaching Hospital, Mazandaran University of Medical Sciences, Sari, Iran; 2 Student Research Committee, Faculty of Medicine, Cardiovascular Research Center, Mazandaran University of Medical Sciences, Sari, Iran; 3 Professor of Cardiology Fellowship of Echocardiography Department of Cardiology Faculty of Medicine, Cardiovascular Research Center Mazandaran University of Medical Sciences, Sari, Iran; 4 Student Research Committee, Faculty of Medicine, Iran University of Medical Sciences, Tehran, Iran

**Keywords:** Atrial, Epicardial adipose tissue, Strain, Speckle-tracking echocardiography

## Abstract

**Background:**

Diabetes mellitus (DM) has been documented among the strongest risk factors for developing heart failure with preserved ejection fraction (HFpEF). The earliest imaging changes in patients with DM are the left atrial (LA) functional and volumetric changes. The aim of this study was to determine the correlation between epicardial fat thickness (EFT) and longitudinal LA reservoir strain (LARS) in patients with type 2 DM (T2DM), as compared with non-diabetic controls.

**Results:**

The study samples in this case-control study comprised of consecutive patients with T2DM (n=64) and matched non-diabetic controls (n=30). An echocardiography was performed on all patients and EFT, volumetric and longitudinal LARS, left ventricular (LV) global longitudinal strain (LVGLS), pulsed-wave Doppler-derived transmitral early (E wave) and late (A wave) diastolic velocities, and tissue-Doppler-derived mitral annular early diastolic (e′) and peak systolic (s') velocities were obtained. The study results demonstrated that the patients with T2DM had thicker EFT (5.96±2.13 vs. 4.10±3.11 mm) and increased LA volume index (LAVI) (43.05± 44.40 vs. 29.10±11.34 ml/m^2^) in comparison with the non-diabetic ones (p-value: 0.005 and 0.022, respectively). On the other hand, a direct association was observed between EFT and the E/e′ ratio, and an inverse correlation was established between EFT and LARS in patients with T2DM (r=0.299, p-value=0.020 and r=− 0.256, p-value=0.043, respectively). However, regression analysis showed only LV mass index (LVMI) (β=0.012, 95% CI 0.006–0.019, p-value<0.001), LAVI (β=− 0.034, 95% CI − 0.05–0.017, p-value<0.001), and EFT (β=− 0.143, 95% CI − 0.264–− 0.021, p-value=0.021) were independently correlated with LARS.

**Conclusions:**

LARS is considered as an important early marker of subclinical cardiac dysfunction. Thickened epicardial fat may be an independent risk factor for decreased LA reservoir strain. Diabetics are especially considered as a high risk group due to having an increased epicardial adipose tissue thickness.

## Introduction

Diabetes mellitus (DM) has been documented among the strongest risk factors for developing heart failure with preserved ejection fraction (HFpEF), a sub-type of heart failure (HF), which represents almost half of the total number of patients with symptomatic HF [1]. Additionally, epicardial adipocyte infiltration has been associated with the mid and late stages of diastolic dysfunction in individuals with type 2 DM (T2DM). In spite of this, the correlation between epicardial fat thickness (EFT) and early dysfunction has not yet been well understood. The paucity of data might be thus attributable to some limitations in the detection of the very early changes in cardiac function [2]. The earliest imaging changes in patients with T2DM are the left atrial (LA) functional and volumetric changes. In this way, such variables can be assumed as useful tools during the early disease detection [3]. As well, T2DM seems to be an independent risk factor for LA enlargement and dysfunction. There is also an association between EFT and LA and left ventricular (LV) functional changes and increased LAVI [2]. The LA contribution to cardiac function is modulated by its effects as the reservoir, conduit, and booster pump function. The reservoir LA function is correlated with LV isovolumic contraction, ejection, and isovolumic relaxation, and is deeply influenced by LV systolic function, atrial size, and compliance. Phasic atrial function can be obtained by the assessment of volumetric or atrial deformation. The volumetric measurement of LA function is, however, limited by its lower sensitivity in an early disease state [4]. LA reservoir strain (LARS) is thus a significant marker of LA dysfunction, which is typically impaired in patients with LV diastolic dysfunction. Elevated LV pressure is associated with the retrograde transmission of pressures into LA, which gradually reduces the LA compliance and impairs atrial relaxation. The final results are reduction in LA reservoir function, LA dilatation, and mechanical failure [5]. The aim of our study was to determine the correlation between EFT and LARS in patients with T2DM and no apparent coronary artery disease (CAD) in comparison with a non-diabetic control group.

## Methods

This study was a case-control study including 64 consecutive patients with T2DM with a left ventricular ejection fraction (LVEF) ≥50% and 30 matched non-diabetic controls, who were hospitalized in our hospital due to evidence of ischemia on non-invasive studies and had no apparent CAD on angiogram between 2021 and 2022. This study was conducted according to the Declaration of Helsinki (DoH) ethical principles, and was further approved by the Ethics Committee of Mazandaran University of Medical Sciences, Sari, Iran (ethics code no. IR.MAZUMS.REC.1400.628). All participants also signed an informed consent form. The patients with significant CAD, HF, severe valvular heart disease, valve replacement, an LVEF<50%, left bundle branch block, atrial flutter or fibrillation, cardiomyopathies, neoplastic illnesses, decreased glomerular filtration rate or serum creatinine ≥1.5 mg/dl, and different systemic diseases were excluded from the study. The serum levels of fasting blood sugar (FBS), cholesterol (Chol), triglyceride (TG), low-density lipoprotein (LDL), and hemoglobin A1c (HbA1c) were determined by the blood sample taken after a 10–12 h overnight fasting. Moreover, 2 h post-prandial serum BS was determined by the blood sample acquired 2 h after eating a meal. The ion exchange chromatography method (Bio Systems S.A, Barcelona, Spain) was used for determining HbA1c level. Body mass index (BMI) was explained as weight in kg divided by height in meters squared. DM was defined according to the guidelines of the American Diabetes Association (ADA), including FBS ≥126 mg/dl (7.0 mmol/L) or 2 h plasma BS during 75 g oral glucose tolerance test (OGTT) ≥200 mg/dl (11.1 mmol/L), or HbA1c ≥6.5% (48 mmol/mol), and included individuals who require to take insulin or oral hypoglycemic medications [6]. Hypertension (HTN) was explained as a systolic blood pressure (SBP) ≥140mmHg and/or a DBP ≥90mmHg, determined on three separate occasions or those receiving antihypertensive medications [7]. Hyperlipidemia (HLP) was characterized as the total Chol levels over 200 mg/dl, HDL-c levels less than 40 mg/dl in males, or less than 50 mg/dl in females [8]. Cigarette smoking was identified by a face-to-face survey. Coronary angiography was performed on all patients by a Siemens AG (Medical Solutions; Erlangen, Germany) within 24–48 h of admission. An experienced cardiologist blinded to the patient data then interpreted the angiograms. No apparent CAD was defined as a stenosis of less than 20% in all coronary artery territories or the presence of only luminal irregularities [9].

### Echocardiography

**Fig. 1:**
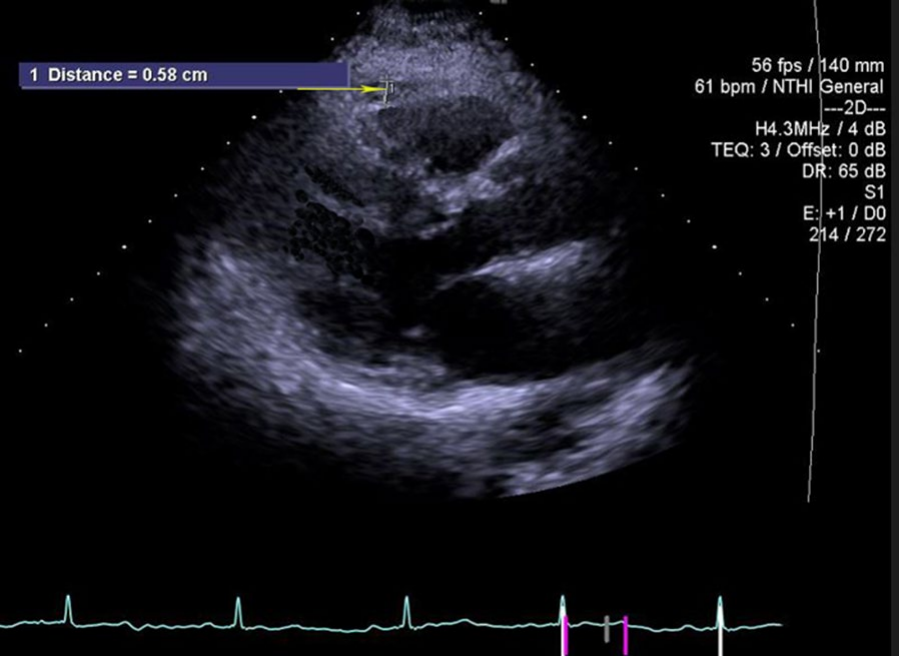
Transthoracic two-dimensional echocardiography in the parasternal long-axis view, showing epicardial fat thickness (arrow)

A transthoracic echocardiography was performed for all patients by the ACUSON SC2000 with a 4V1c transducer (Siemens Medical Solutions Inc., Mountain View, CA, USA) within 24–48 h after hospitalization. All movies and images were stored on a hard disk for further off-line analysis (using the eSie VVI software) by an expert echocardiographer blinded to the patients’data. Epicardial fat was identified as an echo-dense space between the outer wall of the myocardium and the visceral pericardium, anterior to the right ventricular wall in the parasternal long axis view. The point of measurement was vertical to the aortic annulus, and its thickness was measured at the end-systole in an average of three cardiac cycles [10] (Figure 1).

**Fig. 2:**
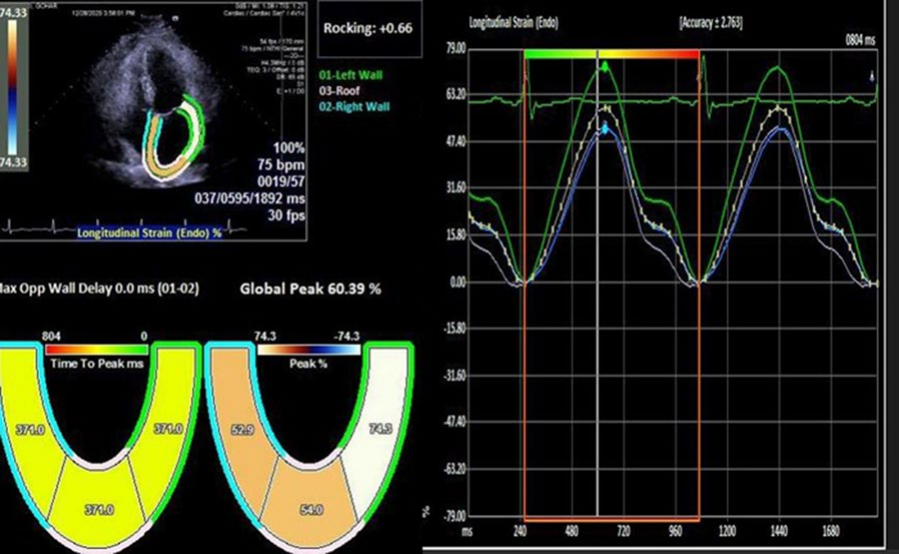
Speckle-tracking echocardiography in apical four chamber view represents longitudinal LA reservoir strain curves in three segments containing left LA wall, roof, and right LA wall by considering the QRS complex (R-R gating) as the initiation of the strain calculation (*LA* Left atrium)

The two-dimensional (2D) grayscale movies were acquired in the apical four-, two-, and three-chamber views (three standard apical views) in three cardiac cycles. The peak longitudinal strain values from the basal, mid, and apical segments of the inferoseptal, anterolateral, inferior, anterior, inferolateral, and anteroseptal walls were measured by the tracking of the endocardial and epicardial walls. To identify the LV peak global longitudinal strain (LVGLS), the average value of all 18 myocardial segments was considered [11]. The LA diameter was identified as the vertical distance between the posterior wall of the aortic root and the posterior LA wall in the para-sternal long axis view at the end systole [12]. The eSie VVI software was used to trace the LA endocardial and epicardial borders in the apical four-chamber view. The average LARS in three segments containing left LA wall, roof, and right LA wall were used to determine LARS by the R-R gating method (Figure 2). After that, LARS was corrected for LVGLS (LARS/-LVGLS). Transmitral pulse-Doppler-derived early and late diastolic velocities (E and A waves) and deceleration time (DT) of the E-wave were determined by placing the cursor at the tip of mitral valve leaflets in the apical four-chamber view. To find the mean tissue-Doppler-derived mitral annulus septal and lateral early diastolic (e°) and peak systolic (s′) velocities, the cursor was inserted at the level of the mitral annulus. The M-mode echocardiography in the parasternal long-axis view was further employed to determine the end-systolic and -diastolic LV internal diameters and end-diastolic interventricular septal (IVS) and posterior wall thickness by inserting the cursor at the mitral valve leaflet tip. The LV mass index (LVMI) was calculated by the formula given below:

$$0.{8}\left( {{1}.0{5 }\left[ {\left( {{\text{LVIDd}} + {\text{PWT}} + {\text{IVST}}} \right){^3}{-} \, \left( {{\text{LVIDD}}} \right){^3}} \right]} \right) + 0.{6}$$

In which, LVIDd, PWT, and SWT indicate the end-diastolic LV internal dimension and posterior and IVS wall thickness, respectively. We also obtained peak velocity of tricuspid regurgitation jet by continuous-Doppler from multiple views [11]. The reproducibility of the EFT and speckle-tracking-derived LVGLS and LARS measurements were determined according to repeated measurements in 10 randomly selected patients by an echocardiography within 48 h, and the intra-observer correlation coefficients were found to be 0.91, 0.90, and 0.92, respectively.

### Statistical analysis

The categorical variables were presented as frequency and percentage. The quantitative variables were expressed as the mean± standard deviation (for the normally distributed continuous variables) or median [lower-upper quartile] (for Non-normally distributed continuous variables). Normality was determined by the Shapiro-Wilk test. An independent t-test was used to compare the continuous variables that were normally distributed and the Mann-Whitney U test was used to compare the continuous variables that were non-normally distributed. The categorical variables were compared by Chi-square test and Fisher’s exact test. Moreover, we used a Pearson correlation to find the correlation between different echocardiographic variables in the patients with T2DM and the non-diabetic ones, separately. Multiple linear regression analysis was also conducted on all study population, including the patients with T2DM and the non-diabetic controls to determine the independent correlation between different demographic, laboratory-based, and echocardiographic variables and LARS/-LVGLS. The statistical analyses were performed by the SPSS/PASW (Predictive Analytics Software) Statistics version 18 (SPSS, Chicago, IL, USA). The P value of less than 0.05 signified that the variable was significant. The sample size was determined based on the findings of Evin et al study [2]. According to mean and SD of LA longitudinal strain in case and control groups of the aforementioned study, the effect size was estimated to be 0.69. Considering a confidence level of 95%, the test power of 80%,, a one-tailed test for the comparison of two means, and using the G*power software, the minimum sample size was determined to be 54 individuals (viz., 27 in each group). Given the available facilities and the aim to increase the power of the statistical tests, more samples (that is, 64 individuals in the case group and 30 people as the controls) were included in this study.

## Results

This study included 64 patients with T2DM and 30 non-diabetic controls, matched in terms of age and gender, with an LVEF ≥50% and no apparent CAD on angiogram. The mean age of the study population was 55.03±9.86 and their mean BMI was 28.14±3.77 kg/m^2^. In total, 71 patients (75.5%) were female, and 23 individuals (24.5%) were male. Moreover, 47 patients (50%) had HLP, 46 people (48.9%) were hypertensive, and 19 individuals (20.2%) were smokers. The demographic variables of the study population are presented in Table 1. As expected, the patients with T2DM had higher FBS, 2 h post-prandial BS, and HbA1c levels in comparison with the non-diabetic controls. Moreover, the TG level and BMI were higher in the patients with T2DM than the non-diabetic ones (p-value: 0.002 and 0.010, respectively). The echocardiographic characteristics of both study groups are depicted in Table 2. The mean EFT in the patients with T2DM was higher than that in the non-diabetic ones (5.96±2.13 vs. 4.10±3.11 mm, p-value=0.005). Furthermore, LAVI, the A-wave velocity, and the E/e’ ratio were higher (p-value: 0.022, 0.023, and 0.001; respectively), and the e’ and s’ velocities were lower (p-value: 0.019 and 0.006, respectively) in the patients with DM, as compared with the non-diabetic ones. The Pearson correlation coefficient was further utilized to determine the correlation between the echocardiographic variables in both groups, separately. The study results also showed a positive correlation between EFT and the E/e’ ratio (r=0.299, p-value=0.020), but a negative correlation between EFT and LARS (r=− 0.256, p-value=0.043) in the patients with T2DM. Such correlations were not seen in the non-diabetic controls (Table 3). To find the independent correlation between different demographic and echocardiographic variables and LARS/-LVGLS, multiple linear regression analysis with backward elimination process was conducted on all study population, including the patients with T2DM and the non-diabetic controls. The result of this analysis showed among different variables, only LVMI (β=0.012, 95% CI 0.006–0.019, p-value<0.001), LAVI (β=− 0.034, 95% CI − 0.05–− 0.017, p-value<0.001), and EFT (β=− 0.143, 95% CI − 0.264–− 0.021, p-value=0.021) had an independent correlation with LARS/LVGLS (Table 4).

**Table 1:** Demographic and laboratory characteristics of the two study groups categorized by having or not having type 2 DM

		Non diabetics (n=30)	Diabetics (n=64)	P value
Age (years)		53.83±10.82	55.59±9.42	0.423
Sex (n, %)	Male	10 (33.3%)	13 (20.3%)	0.171
	Female	20 (66.7%)	51 (79.7%)	
BMI (kg/m^2^)		27.07 [24.98-28.25]	28.38 [26.71-29.72]	0.010
HTN (n, %)		11 (36.7%)	35 (54.7%)	0.103
HLP (n, %)		11 (36.7%)	36 (56.2%)	0.077
Smoking (n, %)		8 (26.7%)	11 (17.2%)	0.286
Heart rate (bpm)		80.00 [66.5-85.00]	78.00 [72.00–84.00]	0.870
FBS (mg/dl)		94 [88-103]	134 [102.50–180]	<0.001
BS2hPP (mg/dl)		126.50 [118.50-133.25]	248.50 [149.00–353.25]	<0.001
TG (mg/dl)		120.57±56.04	176.50±86.97	0.002
Chol (mg/dl)		151.30±40.37	150.03±42.16	0.891
LDL (mg/dl)		88.10±30.37	83.89±31.47	0.543
HbA1c level (%)		5.35 [5.00-5.72]	7.25 [6.62-9.05]	<0.001

*BMI* body mass index, *HTN* Hypertension, *HLP* Hyperlipidemia, *FBS* Fasting blood glucose, *BS2Hpp* serum blood sugar 2 h post-prandial, *TG* Triglyceride, *Chol* Cholesterol, *LDL* Low-density lipoprotein, *HbA1c* hemoglobin A1c

**Table 2:** Echocardiographic characteristics of the two study groups categorized by having or not having type 2 DM

	Non diabetics (n=30)	Diabetics (n=64)	P value
LARS (%)	39.47 [31.67–58.36]	35.61 [24.71–43.59]	0.215
Indexed LA volume (ml/m^2^)	29.10±11.34	43.05± 44.40	0.022
Indexed LA diameter (cm/m^2^)	2.07±0.37	2.51±2.69	0.385
LA EF (%)	68.20±15.28	67.16±15.54	0.762
Indexed LV mass (g/m^2^)	103.01±51.31	117.83±116.23	0.508
Indexed LVIDd (cm/m^2^)	2.75±.39	3.02±2.97	0.624
Indexed LVIDs (cm/m^2^)	1.67±0.29	1.80±1.70	0.684
Epicardial fat thickness (mm)	4.10±3.11	5.96±2.13	0.005
E wave (m/s)	0.62±0.18	0.67±.15	0.229
A wave (m/s)	0.63±0.18	0.73±0.23	0.023
E/A	1.03±0.37	0.96±0.31	0.348
E/e'	6.34±1.65	7.80±2.01	0.001
e’ (cm/s)	10.27±3.04	8.75±2.00	0.019
s’ (cm/s)	9.10±1.40	8.22±1.32	0.006
DT of E wave (ms)	235.43±56.44	227.14±54.22	0.499
LVGLS (%)	− 18.79±3.93	− 16.76±6.33	0.112
TR velocity (m/s)	2.31 [2.07-2.46]	2.36 [2.18-2.51]	0.280
LARS/-LVGLS	2.2903 [1.52-3.32]	1.91 [1.48-3.02]	0.340

*LA* Left atrium, *LV* Left ventricle, *EF* Ejection fraction, *LVIDd* End diastolic left ventricular internal diameter, *LVIDs* End systolic left ventricular internal diameter, *DT* Deceleration time, *GLS* global longitudinal strain, *LA* left atrium, *E/e’* transmitral Doppler early diastolic velocity/mitral annular early diastolic velocity, *A wave* transmitral Doppler late diastolic velocity, *s* mitral annular peak systolic velocity, *TR* tricuspid regurgitation, *LARS* left atrial reservoir strain

**Table 3:** Correlation between different echocardiographic variables in patients with DM and control group

Correlations							
DM			Epicardial fat thickness	LARS	LVGLS	Indexed LV mass	E/e’
DM	Epicardial fat thickness	Pearson Correlation	1	− 0.256	− 0.098	0.092	0.299
		P value		0.043	0.446	0.487	0.020
	LARS	Pearson Correlation	− 0.256	1	0.007	0.016	− 0.034
		P value	0.043		0.954	0.901	0.798
	LVGLS	Pearson Correlation	− 0.098	0.007	1	− 0.044	− 0.152
		P value	0.446	0.954		0.737	0.247
	Indexed LV mass	Pearson Correlation	0.092	0.016	− 0.044	1	− 0.135
		P value	0.487	0.901	0.737		0.318
	E/e'	Pearson Correlation	0.299	− 0.034	− 0.152	− 0.135	1
		P value	0.020	0.798	0.247	0.318	
No DM	Epicardial fat thickness	Pearson Correlation	1	− 0.212	0.293	0.682	0.237
		P value		0.261	0.116	<0.001	0.215
	LARS	Pearson Correlation	− 0.212	1	− 0.190	− 0.075	− 0.104
		P value	0.261		0.314	0.692	0.590
	LVGLS	Pearson Correlation	0.293	− 0.190	1	− 0.066	− 0.079
		P value	0.116	0.314		0.730	0.685
	Indexed LV mass	Pearson Correlation	0.682	− 0.075	− 0.066	1	0.460
		P value	<0.001	0.692	0.730		0.012
	E/e'	Pearson Correlation	0.237	− 0.104	− 0.079	0.460	1
		P value	0.215	0.590	0.685	0.012	

*DM* diabetes mellitus, *LA* left atrium, *LV* left ventricle, *LVGLS* left ventricular global longitudinal strain, *LA* left atrium, *E/e'* transmitral doppler early diastolic velocity/mitral annular early diastolic velocity, *LARS* left atrial reservoir strain

**Table 4:** Multiple linear regression analysis determining independent correlation between different variables with LA longitudinal reservoir strain/LVGLS

Coefficients^a^								
Model		Unstandardized coefficients		Standardized coefficiets	t	P value	95.0% confidence interval for B	
		B	Std. error	Beta			Lower bound	Upper bound
1	(Constant)	4.846	2.766		1.752	0.084	− 0.671	10.364
	Age	− 0.020	0.025	− 0.127	− 0.810	0.420	− 0.070	0.030
	Epicardial fat thickness	− 0.136	0.077	− 0.232	− 1.758	0.083	− 0.290	0.018
	DM	− 0.083	0.431	− 0.026	− 0.192	0.848	− 0.943	0.777
	Indexed LV mass	0.016	0.004	1.048	3.784	<0.001	0.007	0.024
	BMI	− 0.016	0.048	− 0.041	− 0.337	0.737	− 0.112	0.080
	E/A	− 0.227	0.832	− 0.050	− 0.273	0.785	− 1.887	1.432
	E/e’	0.065	0.113	0.087	0.577	0.566	− 0.160	0.290
	HTN	− 0.601	0.370	− 0.197	− 1.628	0.108	− 1.339	0.136
	TG	0.002	0.002	0.126	1.081	0.283	− 0.002	0.007
	Chol	− 0.004	0.004	− 0.099	− 0.916	0.363	− 0.012	0.004
	Indexed LA volume	− 0.041	0.011	− 1.057	− 3.794	<0.001	− 0.063	− 0.019
	e ‘	0.123	0.131	0.195	0.936	0.353	− 0.139	0.385
	S’	− 0.058	0.146	− 0.053	− 0.398	0.692	− 0.350	0.233
11	(Constant)	3.060	0.361		8.470	<0.001	2.341	3.779
	Epicardial fat	− 0.143	0.061	− 0.243	− 2.338	0.022	− 0.264	− 0.021
	Indexed LV mass	0.012	0.003	0.832	3.765	<0.001	0.006	0.019
	Indexed LA volume	− 0.034	0.008	− 0.863	− 3.969	<0.001	− 0.050	− 0.017

*DM* diabetes mellitus, *BMI* body mass index, *HTN* hypertension, *TG* Triglyceride, *Chol* Cholesterol, *LA* Left atrium, *LV* Left ventricle, *E/e*’: transmitral doppler early diastolic velocity/mitral annular early diastolic velocity, *A wave* transmitral doppler late diastolic velocity, *LARS* left atrial reservoir strain, *LVGLS* left ventricular global longitudinal strain

^a^Dependent Variable: LARS/LVGLS

## Discussion

LAVI represents the long-standing effects of increased LV filling pressures [13]. LARS is a stronger predictor of outcome than LAVI and LV parameters [14]. Some studies have validated the prognostic and diagnostic values of LARS in the early detection of some cardiovascular diseases (CVDs) [15]. A powerful correlation exists between LAVI and its mechanical function, but alterations in LA deformation may frequently happen before cavity remodeling and enlargement, and LARS can be thus an important early marker of subclinical cardiac dysfunction [16]. In the atrial reservoir phase, LA is filled and stretched, and there is a positive atrial strain that reaches its peak during the LV systole. Decreased LARS is also a significant, independent predictor of LA fibrosis and remodeling [17]. The normal reference range for LARS is 39.4% (95% CI 38.0–40.8%) [4]. Kurt et al. reported that the patients with HFpEF had lower LARS as compared with those with LV diastolic dysfunction with no HF. They also reported that those with LARS *<*23% had worse New York Heart Association (NYHA) functional class and higher pulmonary capillary wedge pressure (PCWP) in comparison with those with a higher value [18]. LA also acts as a reservoir during the LV systole. The LA structural and functional changes are considered as the earliest imaging changes in patients with obesity and T2DM [2]. Since LARS could be strongly influenced by LV systolic function, it was corrected for LVGLS (LARS/-LVGLS) to better represent the LA function for any LV shortening, and demonstrate left atrioventricular interaction [19]. Previous studies have established a correlation between HbA1c level and mean BS level over the past three months [20]. For this reason, this variable was included in the present study. On the other hand, EFT is an active visceral adipose tissue located between the visceral pericardium and myocardium. In addition to its physiological roles, it can also lead to the secretion of multiple pro-inflammatory cytokines, and is correlated with metabolic syndrome pathogenesis and insulin resistance [21]. Its thickness can be simply and perfectly determined by standard echocardiographic views [10]. Elevated EFT is usually associated with metabolic syndrome and is considered as a risk marker of CVDs [22]. The correlation between EFT and T2DM has been also validated [23], and individuals with impaired FBS usually have thicker EFT in comparison with normoglycemic cases [24].

In 2018, Zhao et al conducted a prospective study on 130 people without CAD or atrial fibrillation. Indexed epicardial adipose tissue (EAT) volume was determined by cardiac computed tomography (CT). As well, three-dimensional (3D) volumetric measurements and 2D speckle-tracking echocardiography were performed. The study population were then divided into two groups of large and small EAT volume index. The individuals with larger EAT volume index had significantly impaired LAEF and longitudinal LARS. The total EAT volume index could be thus a predictor of impaired longitudinal LARS and reduced LAEF. They concluded that there was an independent association between EAT volume index and subclinical LA dysfunction [17]. Their findings were accordingly consistent with the results in the present study that EFT was an independent predictor of decreased LARS. In 2016, Evin et al. performed a study on 20 patients with obesity and T2DM and 19 healthy controls to assess the association between cardiac adipose tissue and LA function. Cardiac magnetic resonance imaging (MRI) data had been then acquired on all patients to determine systolic LV size and function, longitudinal LARS and strain rates, and radial motion fraction and velocities. They found that epicardial fat volume (EFV) was significantly higher in the patients with obesity and T2DM than controls. Moreover, there were significant correlations between LA functional parameters and both BMI and EFV [2], which were in agreement with the present study that the patients with T2DM had thicker EFT and there was an independent correlation between EFT and decreased LARS.

Mitral annular motion plays an important role in regulating blood movement in and out of LA. During systole, the mitral annulus descends and transfers more apically. These descending motions can further accelerate LV ejection. In addition, this motion can enlarge LA and increase its compliance, accompanied by a drop in LA pressure and a rise in the pulmonary blood flow during atrial reservoir phase [25]. Delayed relaxation further reduces the early diastolic transmitral gradient, and limits the early diastolic filling, which induces an increase in blood residue in LA. As a result, more blood should be ejected into LV during the atrial systole, which is associated with increased LA stroke volume and the peak A-wave velocity [26]. In the present study, the patients with T2DM had lower tissue-Doppler-derived mitral annular s' velocity and higher pulse-Doppler derived A-wave velocity, as compared with the non-diabetics.

During the rapid filling phase, LV is relaxed and mitral annulus ascends and gets away from the apex. The resultant drop in the LV pressure then opens the mitral valve and intensifies ventricular filling (in the atrial conduit phase) [27]. Previous studies have suggested that the E/e' ratio is helpful in estimating the mean LA pressure [28]. Indeed, E wave can be considered as a substitute for the LA-LV pressure gradient and the e' velocity indicates the extent of the gradient created by ventricular suction (29). Our patients with T2DM had larger LA volume, lower tissue-Doppler-derived mitral annular e' velocity, and higher E/e' ratio than the non-diabetics. These findings represent higher filling pressure and diastolic dysfunction in patients with T2DM compared with the non-diabetic ones.

## Conclusion

LARS is considered as an important early marker of subclinical cardiac dysfunction. Thickened epicardial fat may be an independent risk factor for decreased LA reservoir strain. Diabetics are especially considered as a high risk group due to having an increased epicardial adipose tissue thickness.

## Limitations

There were some limitations in this study, including the small sample size recruited in a single center. As well, the echocardiography, which is a relatively inexpensive and easily accessible modality, was used to determine EFT. However, measurement of the EFT volume by the CT scan and MRI techniques could lead to more accurate results. Moreover, LA volume and reservoir strain, but not the other components of LA function, were measured in this study. On the other hand, information on patient therapy, time since diabetes diagnosis, and creatinine values were not included for data analysis. In spite of this, the HbA1c level that could be correlated with the mean BS concentration over the past three months was included in this study.

## Data Availability

The datasets used and/or analysed during this study are available from the corresponding author on reasonable request.
